# Do we need to adjust for interim analyses in a Bayesian adaptive trial design?

**DOI:** 10.1186/s12874-020-01042-7

**Published:** 2020-06-10

**Authors:** Elizabeth G. Ryan, Kristian Brock, Simon Gates, Daniel Slade

**Affiliations:** Cancer Research UK Clinical Trials Unit, Institute of Cancer and Genomic Sciences, University of Birmingham, Birmingham, UK

**Keywords:** Bayesian, Adaptive design, Type I error, Interim analysis, Multiple comparisons, Randomised controlled trial, Multiplicities

## Abstract

**Background:**

Bayesian adaptive methods are increasingly being used to design clinical trials and offer several advantages over traditional approaches. Decisions at analysis points are usually based on the posterior distribution of the treatment effect. However, there is some confusion as to whether control of type I error is required for Bayesian designs as this is a frequentist concept.

**Methods:**

We discuss the arguments for and against adjusting for multiplicities in Bayesian trials with interim analyses. With two case studies we illustrate the effect of including interim analyses on type I/II error rates in Bayesian clinical trials where no adjustments for multiplicities are made. We propose several approaches to control type I error, and also alternative methods for decision-making in Bayesian clinical trials.

**Results:**

In both case studies we demonstrated that the type I error was inflated in the Bayesian adaptive designs through incorporation of interim analyses that allowed early stopping for efficacy and without adjustments to account for multiplicity. Incorporation of early stopping for efficacy also increased the power in some instances. An increase in the number of interim analyses that only allowed early stopping for futility decreased the type I error, but also decreased power. An increase in the number of interim analyses that allowed for either early stopping for efficacy or futility generally increased type I error and decreased power.

**Conclusions:**

Currently, regulators require demonstration of control of type I error for both frequentist and Bayesian adaptive designs, particularly for late-phase trials. To demonstrate control of type I error in Bayesian adaptive designs, adjustments to the stopping boundaries are usually required for designs that allow for early stopping for efficacy as the number of analyses increase. If the designs only allow for early stopping for futility then adjustments to the stopping boundaries are not needed to control type I error. If one instead uses a strict Bayesian approach, which is currently more accepted in the design and analysis of exploratory trials, then type I errors could be ignored and the designs could instead focus on the posterior probabilities of treatment effects of clinically-relevant values.

## Background

The type I error rate, which is the probability of rejecting the null hypothesis when no difference truly exists between treatment arms, is often an important quantity in randomised controlled trials (RCTs). When null hypotheses are repeatedly tested they will eventually be rejected with a probability of 1, and thus the probability of making a type I error increases. Multiplicities can arise in RCTs by testing multiple hypotheses (e.g., multiple endpoints, treatment group comparisons, or subgroup analyses) or from repeatedly testing the same hypothesis over time (e.g., sequential designs). Here we focus on the latter where multiplicities arise from performing interim analyses on accumulating data in an RCT.

Demonstration of the control of type I error at a pre-specified level is generally a prerequisite for a clinical trial design to be accepted by regulators, particularly for late phase trials. Frequentist adaptive designs, such as group sequential designs, typically perform corrections to the stopping boundaries to ensure that the overall type I error rate is maintained at some specific level, e.g., 5% (see [1, 2]). The statistical theory is well developed to control the type I error rate for frequentist adaptive designs.

Bayesian methods are increasingly being used to design adaptive trials (e.g., [3–5]). Rather than making inference by using *p*-values calculated from test statistics within the null hypothesis testing framework, Bayesian approaches can instead focus on the posterior probability of clinically relevant treatment effect values, for example, the probability the relative risk (RR) is < 1. In Bayesian adaptive designs, efficacy and futility criteria can be based on the probability of treatment effects, given the observed data. For example, one could stop for efficacy if the posterior probability that the hazard ratio (HR) was < 1 was above 90%, i.e., Pr (HR < 1|data) > 0.9. Similarly, one could stop for futility if Pr (HR < 1|data) < 0.1.

Those who routinely work with Bayesian adaptive designs, particularly in later phase trials, will be familiar with the regulatory requirements and approaches that can be used for type I error control for Bayesian adaptive designs. (e.g., [6–9]). However, some confusion remains amongst statisticians newer to these approaches and trialists/clinicians as to whether control of type I error is required for Bayesian adaptive designs, since this is typically considered a frequentist concept, and whether any adjustments need to be made to account for interim analyses/multiplicities. The aim of this work is to clarify if and when adjustments are required in Bayesian adaptive designs to control type I error by accounting for the multiple analyses that may be performed and decisions made. We will also use several illustrative case studies to show how the performance of Bayesian adaptive designs can be affected by incorporating interim analyses, in terms of their “type I error” and “power”, when the stopping boundaries are not adjusted to account for multiple analyses. In this work we will only address multiplicities that arise from interim analyses where a single outcome is repeatedly measured over the course of an RCT.

## Methods

### Current practice for Bayesian RCTs

The Bayesian approach, which is conditional on the data observed, is consistent with the strong likelihood principle. The final analysis can ignore the results and actions taken during the interim analyses and focus on the data actually obtained when estimating the treatment effect (see, for example, [10, 11]). That is, *inferential* corrections, e.g., adjustments to posterior probabilities, are not required for multiple looks at the data and the posterior distribution for the parameter of interest can be updated at any time. This is appealing to clinicians who are often confused about why previous (or multiple concurrent in the case of multiple arms/outcomes) inspections of the trial data affect the interpretation of the final results in the frequentist framework where adjustments to *p*-values are usually required. The stopping rule in a Bayesian adaptive design does not play a direct role in a Bayesian analysis, unlike a frequentist analysis. One can also calculate several probabilities from the posterior without the need for adjustment for multiple comparisons, such as: the probability RR < 0.5, 0.75, 0.9, 1 and the probability that the absolute risk difference is < 0. In decision making however, the Bayesian approach can be just as vulnerable to multiplicities through performing interim analyses as the frequentist approach for controlling type I error [12]. Corrections may be required to the Bayesian stopping boundaries in the design phase to assist with decision making and demonstrate that the Bayesian design has good frequentist operating characteristics (i.e., high power and acceptable type I error).

Whilst the long-run frequency behaviour of sequential testing procedures is irrelevant from the strict Bayesian perspective, long-run properties have been established as being important in the clinical trial setting, particularly for late phase trials, as there can be high costs to making incorrect decisions [6, 13, 14]. Confirmatory trials will have to persuade, amongst the normal sceptical scientific audience, the competent authorities for healthcare regulation. Depending on the jurisdiction, this may include the European Medicines Agency (EMA), the US Food and Drug Administration (FDA), or the Medicines and Healthcare products Regulatory Agency (MHRA). One of the core functions of health-care regulators is to prevent those that market interventions from making spurious claims of benefit. For this reason, adequate control of type I error is one of the perennial concerns when appraising the results of confirmatory clinical trials. Thus in confirmatory trials, demonstration that the type I error is controlled is likely to be required.

Simon [7] acknowledged that multiplicity issues are of importance in both the frequentist and Bayesian frameworks, and that problems in operating characteristics do not disappear by using Bayesian methods. Freedman and Spiegelhalter [8] also note, *“More frequent applications of the Bayesian rule during the trial would, however, change the frequentist properties of the monitoring scheme.”* Depending on the choice of design or stopping boundaries, Bayesian interim monitoring may violate the weak repeated sampling principle [15], which states that, *“We should not follow procedures which for some possible parameter values would give, in hypothetical repetitions, misleading conclusions most of the time”*. Thus, using these views, the Bayesian designs must be chosen by considering problems of multiplicity resulting from sequential monitoring of accumulating results.

In the clinical trial setting Bayesian inference is often mixed with non-Bayesian decision making. Decisions at the analyses are usually made by comparing some summary of the accumulated data, such as the posterior probability that the treatment effect exceeds a particular value, to a pre-specified boundary. For example, a Bayesian adaptive trial could allow for early stopping for efficacy or futility. At each interim analysis, the posterior probability of having a RR < 1 (indicating benefit) is calculated based on the accumulated data. If that probability is sufficiently high, i.e., above a pre-specified threshold value *b*_*u*_, then the trial may stop early for efficacy. If the probability is very low, i.e., below a pre-specified value *b*_*L*_, then the trial may stop early for futility. If the probability falls between these two values *b*_*L*_ and *b*_*u*_ then the trial may continue recruiting. Clinicians, statisticians and funders will often want to know how often a given design will conclude in favour of a particular treatment, or the effect of a given stopping rule on the operating characteristics.

Although the type I error and power are frequentist concepts by definition, we can calculate something analogous to these quantities for any pre-specified decision rule, whether it is frequentist or Bayesian. Many Bayesian adaptive trial designs are required by funders and regulatory agencies to demonstrate adequate control of error rates (e.g., [16, 17]). For some Bayesian designs with certain prior and likelihood combinations the type I error can be computed analytically. In practice however, Bayesian designs usually rely on simulations to calculate the type I error. This is achieved by determining how frequently the Bayesian design incorrectly declares a treatment to be effective or superior when it is assumed that there is truly no difference, for the given decision criteria/stopping boundaries. The power for a specific treatment effect can be calculated as the proportion of simulations that declare the trial to be “successful” based on the given decision criteria when the target treatment effect is assumed to be the true value. This approach has been recommended by the FDA [17] and has been used in practice for Bayesian adaptive designs (e.g., [18, 19]). These simulations should be performed in the planning stage of a Bayesian adaptive design and corrections may be made to the stopping boundaries if required during the design process. In the analysis stage of the Bayesian adaptive design, no further adjustments are required to account for the previous (interim) analyses that have been performed.

If one wishes to control error rates, such as the probability of declaring a treatment arm to be superior when there is truly no difference between the treatments, then choice of stopping boundaries is important in Bayesian adaptive designs. This was demonstrated by Rosner and Berry [6], in which they stated, *“If one feels uncomfortable with the number of rejections, especially early in the trial, one might consider varying the posterior probability required for stopping accrual at each interim analysis.”* Simulation-based approaches have often been used to tune stopping boundaries in Bayesian adaptive designs to ensure acceptable type I error, e.g., 2.5% 1-sided type I error or 5% 2-sided type I error (see for example [18–21]). Theoretical and numerical approaches have also been proposed to find the optimal stopping boundaries for Bayesian adaptive designs which control the type I error through use of alpha-spending functions (e.g., [8, 9, 22–24]).

Below we use two simulations studies to demonstrate the effect of performing an increasing number of interim analyses on the operating characteristics of Bayesian adaptive designs when no adjustments are made to the stopping boundaries. The effect of an increasing number of interim analyses has previously been shown (e.g., [6, 17]). These results are intended to be illustrative examples to support our arguments.

### Case study 1 – time-to-event outcome

Consider a two-arm RCT with one Bayesian analysis where the planned approach is to produce a posterior distribution for a HR. Using a normal-normal conjugate analysis for the log HR, we can obtain an expression for the posterior distribution of the log HR directly without the need to simulate data from some time-to-event distribution. Thus, we can sample log HRs from an assumed normal distribution. We assume a target HR = 0.7 with 200 total events required.

Now, consider a trial decision criterion for success of Pr(*HR* < 1| *Data*) > 0.9. That is, we require that the posterior probability that the HR < 1 is greater than 0.9 at the final analysis (at 200 events) for the trial to be declared successful.

Initially we explore the operating characteristics of a design that does not perform any interim analyses. We then add in an interim analysis at half of the observed events (i.e., 100 events) in which the trial may stop early for futility if Pr(*HR* < 1| *Data*) < 0.5. That is, we require that the probability that the HR < 1 is greater than 0.5 to continue with the trial. The same decision criteria for trial success (as above) is used at the final analysis (if the trial continues to 200 events).

Then we consider to instead have an interim analysis at half of the observed events in which the trial may stop early for superiority of the intervention if Pr(*HR* < 1| *Data*) > 0.9. We use the same criteria at the analysis at 200 events (if the trial does not stop early) (as above) for determining whether the trial was successful.

The operating characteristics for each design were obtained via simulation using a custom written script in R (version 3.6), which is available in Additional file 1. We consider a ‘null’ scenario of HR = 1 as well as HRs of 0.9, 0.8 and 0.7. We simulate 1 million trials for each true HR. We count the number of trials that were declared to be successful. “Successful” trials are those that meet the decision criteria for success mentioned above; these could be trials that stopped at the interim analysis for superiority or continued recruiting until 200 events had been observed. The proportion of trials that were successful when assuming a HR = 1 provide the simulated type I error rate. The power is provided by the proportion of successful trials when HR = 0.7 was assumed. For simplicity, a vague prior was used for the log HR, which had a normal distribution with a mean of 0 and a variance of 10,000.

### Case study 2 – binary outcome

Here we consider incorporating interim analyses into the Beta Agonist Lung injury TrIal 2 (BALTI-2 [25]) which compared the effect of intravenous infusions of salbutamol with placebo on 28-day mortality in patients with acute respiratory distress syndrome (ARDS). The BALTI-2 trial had a target sample size of 1334 patients (667 each arm). The trial assumed a control arm rate of 44% and aimed to detect a 9% reduction in 28-day mortality with 90% power. A two-sided significance level of 0.05 was used and 3% dropout was assumed.

In the Bayesian designs we are interested in incorporating interim analyses that allow early stopping for efficacy and/or futility and will examine the impact that an increasing number of interim analyses has on the designs’ operating characteristics. Since we are interested in demonstrating superiority of the intervention over the control, the designs will be constructed as one-sided superiority studies with a target type I error of 2.5%. We define a “successful” trial as one which declares superiority of the intervention with a high probability.

**Fig. 1:**
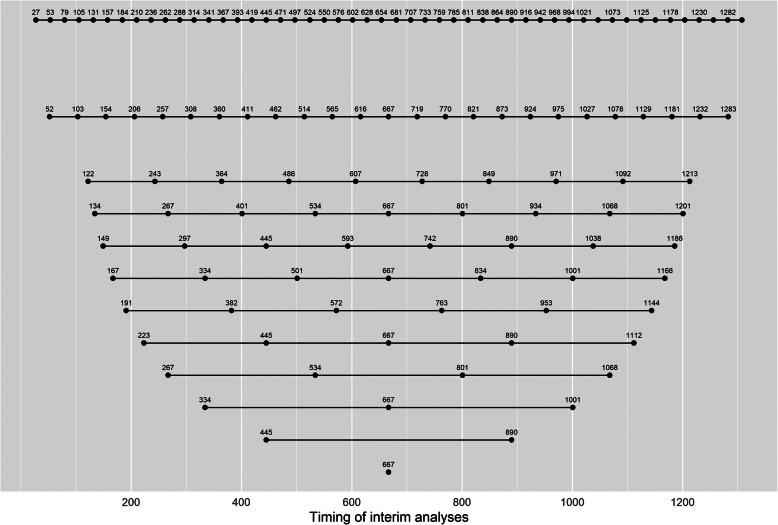
Timings of the interim analyses for the Bayesian designs for the binary outcome case study. The interim analyses occurred once a certain number of patients had been recruited. Each row represents a different design

We began by exploring the operating characteristics of a fixed design with no interim analyses. We then examined the operating characteristics of designs which had between 1 and 10 interim analyses that were evenly spaced by the number of patients recruited; see Fig. 1 and Additional file 2 for the timings of the analyses. We also looked at designs with 25 and 50 interim analyses (evenly spaced by the number of patients recruited) to explore the limiting behaviour of the type I error and power. We explored designs that allowed for early stopping for efficacy only; early stopping for futility only; and either early stopping for efficacy or futility.

Stopping early for efficacy was based on the posterior predictive probability of trial success at the current sample size, after accounting for uncertainty in enrolled patients that had not completed follow-up. This is denoted by P_curr_. The trial was stopped early for efficacy if P_curr_ > 0.99. Stopping early for statistical futility was based on the posterior predictive probability of trial success at the maximum sample size, which is denoted by P_max_. We assumed the maximum sample size was the target sample size in the original frequentist trial design (*N* = 1334). The trial was stopped early for futility if P_max_ < 0.10. The same stopping boundaries were used at each interim analysis. Patients who had not completed their 28-day follow-up at an interim analysis had their response simulated from the posterior distribution.

At the final analysis the trial was declared successful if the posterior probability that the intervention arm has a lower 28-day mortality rate is > 0.98, i.e., Pr(*θ*_*intervention*_< *θ*_*control*_ ∣ *data*) > 0.98, where *θ*_*intervention*_ and *θ*_*control*_ are the 28-day mortality rates in the intervention and control arms, respectively. Otherwise the trial was not successful.

The operating characteristics were obtained by simulating trials in the Fixed and Adaptive Clinical Trial Simulator (FACTS) program (version 6.2 [26]). For each design we simulated 10,000 trials, assuming particular true 28-day mortality rates for each arm. The type I error was calculated from the simulations under the null hypothesis scenario of no difference (assuming 44% 28-day mortality in both arms), and was estimated as the proportion of such simulations that falsely declared the intervention superior. The power was calculated as the proportion of simulations that concluded that the intervention was superior under the target difference of 9%. For simplicity, we used non-informative prior distributions for the primary outcome rate for each arm that were essentially a uniform distribution.

## Results

### Case study 1 – time-to-event outcome

**Fig. 2:**
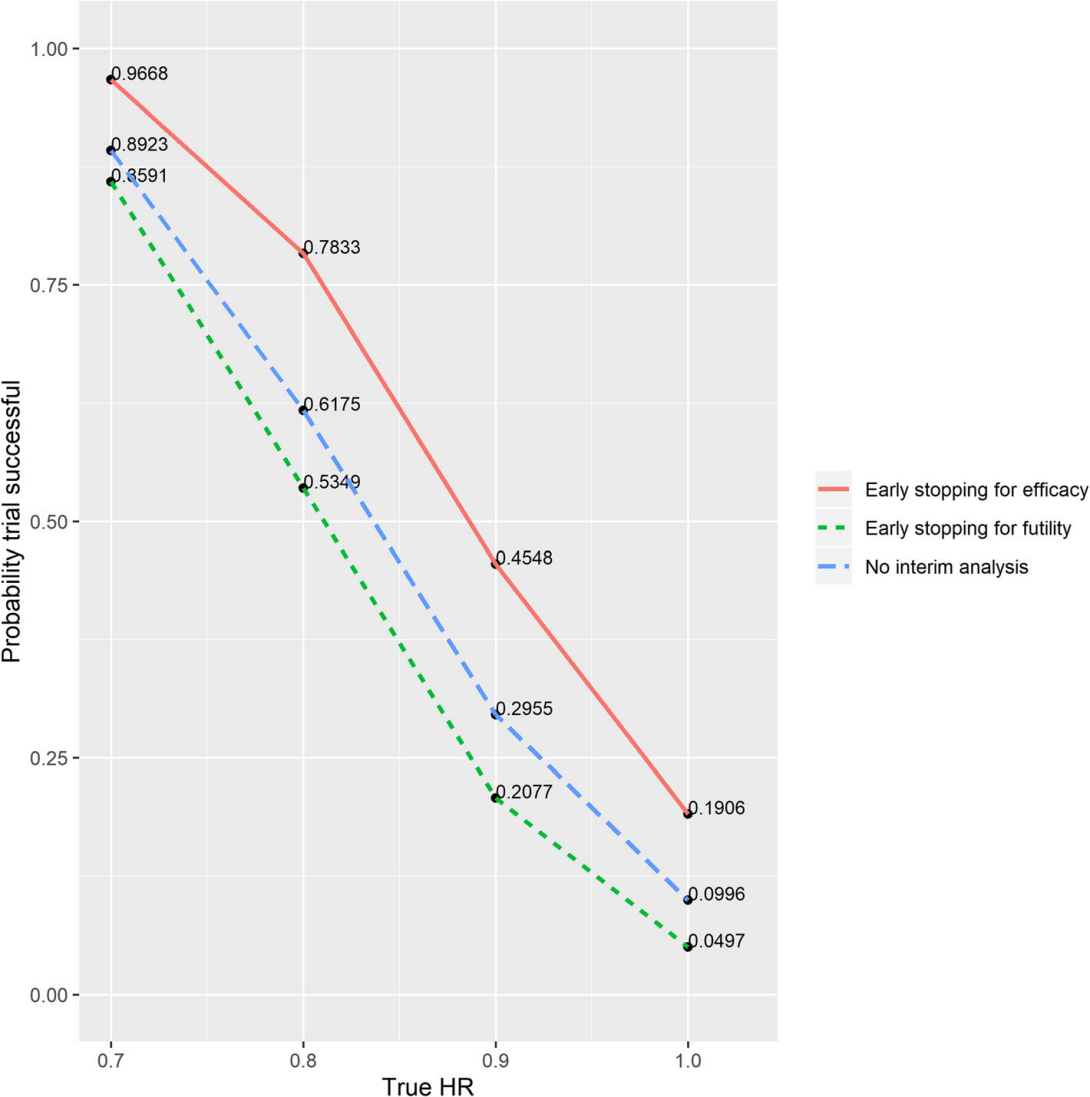
Proportion of trials declared successful under a range of true hazard ratios (HR). The designs explored had no interim analysis; 1 interim analysis that allowed early stopping for futility only; 1 interim analysis that allowed early stopping for efficacy only

The operating characteristics for each design are given in Fig. 2. The type I error rate is reduced by including an interim analysis to assess for futility; however the type II error rate also increased resulting in a lower power for the trial. When early stopping for efficacy was included in the design the type I error rate increased, but the power also increased. Thus through this simple example, the impact of incorporating an interim analysis on the operating characteristics is demonstrated.

For the design that only allows early stopping for efficacy, if we instead use more stringent efficacy stopping boundaries we can reduce the type I error rate. For example, if we allow stopping for efficacy at the interim analysis if Pr(*HR* < 1| *Data*) > 0.95 and also use this criteria at the final analysis (at 200 events) for declaring the trial to be successful, then the type I error decreases to 9.8% (from 19%).

### Case study 2 – binary outcome

**Fig. 3:**
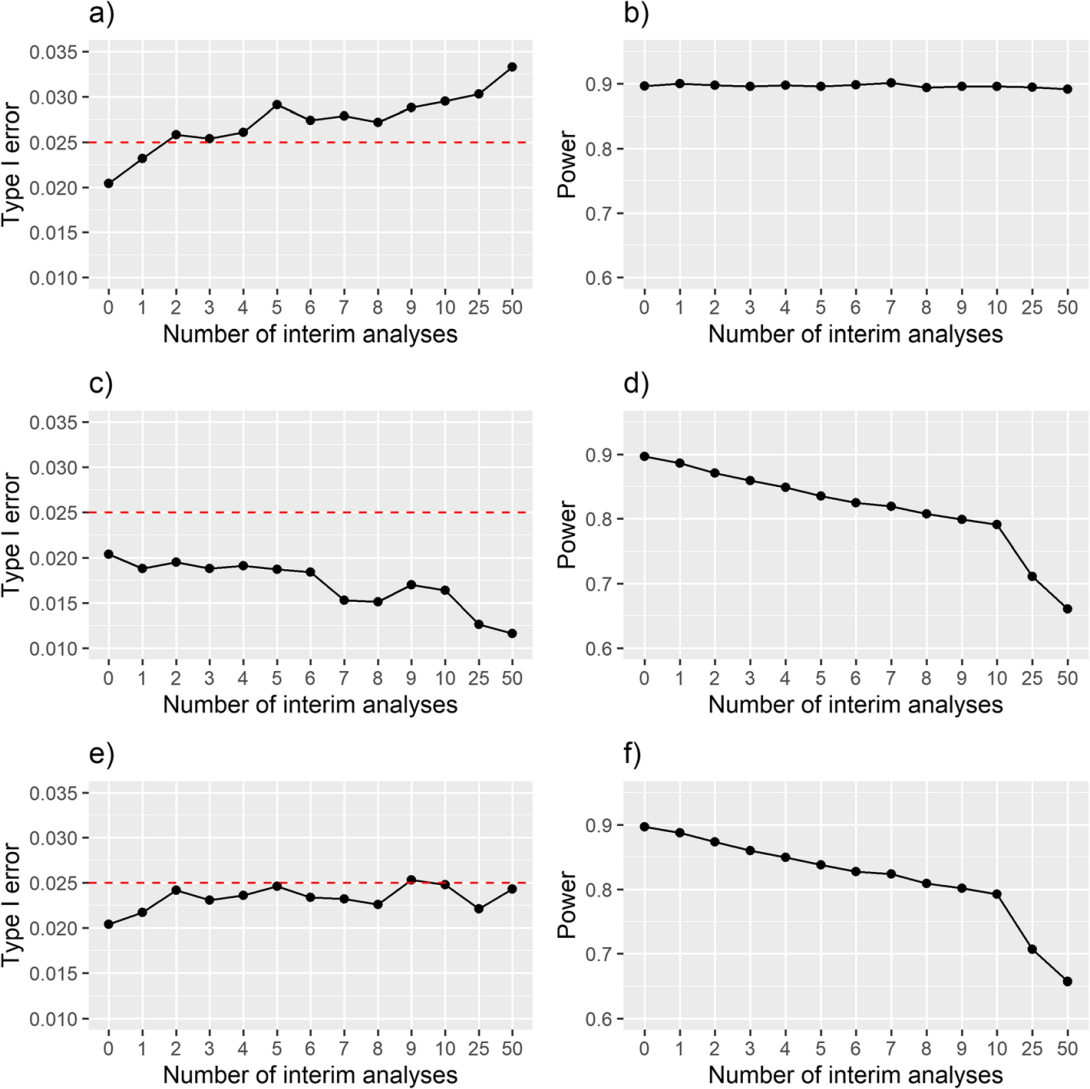
Type I error and Power for Bayesian sequential designs for the binary outcome example: **a** and **b** are for designs that only allow early stopping for efficacy; **c** and **d** are for designs that only allow early stopping for futility; **e** and **f** are for designs that allow early stopping for either efficacy or futility. The dotted horizontal line in **a**, **c** and **e** represents a type I error of 2.5%. The type I error (left column) was calculated assuming both arms had a 28-day mortality of 44%; the power (right column) was calculated assuming 28-day mortality of 35% and 44% in the intervention and control arms, respectively

The operating characteristics for each design are presented in Fig. 3 and Table A2 of Additional file 2. Without adjustment of the stopping boundaries, the type I error increased above the desired level of 2.5% as more interim analyses were included that allowed early stopping for efficacy only (Fig. 3a); the power had little variation with the number of interim analyses for these designs (Fig. 3b). When early stopping for futility was instead permitted at the interim analyses, the type I error rate generally decreased (Fig. 3c), as well as the power (Fig. 3d). When both early stopping for efficacy or futility were permitted in the designs, the type I error generally increased, but fluctuated with the number of interim analyses (Fig. 3e); the power generally decreased for these designs (Fig. 3f).

## Discussion

Here we have shown through two illustrative examples how a Bayesian adaptive design can inflate the type I error rate through incorporation of interim analyses that allow early stopping for efficacy and do not make adjustments to account for multiplicity. Incorporation of early stopping for efficacy also increased the power in some instances. We also showed that not all actions that may be taken at interim analyses in Bayesian adaptive designs cause inflation of type I error: interim analyses that only allowed early stopping for futility decreased the type I error. It is generally early stopping for efficacy that can inflate the type I error during the multiple analyses of adaptive designs. Therefore, we need not be concerned with an increasing type I error with the number of interim analyses if they do not allow for early stopping for efficacy. However, for designs that only allowed early stopping for futility, an increase in the number of interim analyses led to a decrease in power. Whilst these results are not new we hope to provide some clarity on the scenarios in which adjustments need to be made to stopping boundaries in Bayesian adaptive designs to control for type I error.

Dmitrienko and Wang [27] and Berry et al. [14] demonstrated that use of a sceptical prior (centred on a treatment difference of 0) can decrease the type I error since it “pulls back” the posterior mean if you stop early for efficacy. Use of sceptical priors has also been recommended by Spiegelhalter et al. [28] to provide Bayesian designs with good frequentist properties. Whilst informative priors are useful in smaller early phase studies, where higher alpha levels are generally permitted, they are rarely used in confirmatory trials since they can lead to increased type I error rates if additional adjustments are not made to the stopping boundaries (e.g., [14, 23]). Kopp-Schneider et al. [29] demonstrated that strict control of type I error implies that no power gain is possible under any mechanism of incorporation of prior information. Informative priors that favour an intervention represent a clash in paradigms between the role of the prior in a Bayesian analysis and type I error control in the frequentist framework (which requires an assumption of zero difference). Type I errors are unlikely to be of interest to strict Bayesians, particularly if there is evidence for a non-zero effect that is represented in an informative prior.

It has also been recommended that not having interim analyses “too early” can assist in controlling type I error (e.g., [30, 31]). For example, in the binary case study above, if one used a design that only allowed for early stopping for efficacy with three interim analyses that were performed at 600, 900 and 1100 patients instead of 334, 667, 1001 patients (as above), then the type I error is reduced from 2.54 to 2.43%. One could also control for type I error in designs that allow for early stopping for efficacy by using more stringent stopping boundaries for earlier interim analyses, as we demonstrated in the HR case study. Shi and Yin [23] showed that through careful calibration of the stopping boundaries, Bayesian sequential designs can maintain the frequentist type I and II error rates at the nominal levels.

Bayesian stopping rules that do not involve dichotomous decisions, such as sampling until the width of a credible interval reaches a certain value, do not suffer from the multiple testing problem of adaptive designs and one can keep on adding observations until the criteria are met [32]. In this instance, the designer would need to show that the posterior probabilities are well calibrated.

### An alternative approach – beyond type I errors

Bayesians have often argued that use of type I and II errors are inappropriate since they address the wrong question and/or are frequentist concepts. Spiegelhalter et al. [13] notes, *“From a Bayesian perspective control of type I error is not central to making valid inferences and we would not be particularly concerned with using a sequential scheme with a type I error that is not exactly controlled at a specified level.”* The requirement of type I error control for Bayesian adaptive designs causes them to lose many of their philosophical advantages, such as compliance with the likelihood principle, and creates a design that is inherently frequentist (see [14]). Frequentists calculate the probability of data, not the probability of an unknown parameter (e.g., treatment effect), and so the more looks you have the more opportunities you have for data to be extreme. Bayesians do not calculate the probability of data, they calculate the probability of efficacy, and so repeated analyses should not impact the probability of efficacy. Given the recent discussions to abandon significance testing [33, 34] it may be useful to move away from controlling type I error entirely in trial designs.

Instead, decisions may be based purely on the posterior (predictive) probabilities of treatment effects and decision boundaries may be chosen using clinically important values for subsequent decision making (rather than those that produce suitable type I error; e.g., [28]). Berry [35] used posterior probabilities for monitoring without formally pre-specifying a stopping criterion or sample size. “Type I errors” or incorrect decisions can arise in the Bayesian setting through the dichotomisation of trial results into being “successful” or “negative”, for instance, by using a binding decision rule at the final analysis for declaring efficacy of the intervention. If we avoid this dichotomisation and simply report the posterior probability of benefit, then we could potentially avoid having to specify the type I error of a Bayesian design.

Bayesians have argued that type I errors aren’t the most interesting or important type of error, and that we should be more interested in the posterior probability that the treatment is not effective (e.g., [36]). Posterior distributions provide a means to estimate the value of the parameter of interest, conditional on the observed data, and from this we can obtain a probability of efficacy. Type I errors calculate the probability of data conditional on some assumed fixed value of the parameter of interest (e.g., treatment effect = 0), which is unlikely to ever occur *exactly*. It does not give the probability of a decision maker’s regret.

Berry et al. [14] acknowledge that in the future trials may be evaluated using fully Bayesian notions of utilities and decisions. A fully Bayesian decision-making approach uses a utility function to determine the actions to take at each analysis to best achieve the aim of the trial. That is, you choose the decision with the highest expected utility. The Bayesian decision theoretic approach involves averaging over the parameter space and the unobserved data, rather than assuming a “true” fixed value for the unknown parameters of interest. The utility function may contain trade-offs that occur when taking a particular action, such as cost-effectiveness or efficacy-toxicity, and so a fully Bayesian decision-theoretic approach (that uses a Bayesian utility function) does not require error control of a decision boundary in the traditional sense of type I/II error rates. However, these approaches are often computationally intensive and have not often been used in practice (e.g., [37, 38]). Spiegelhalter and Freedman [39] argue that implicit recognition of the costs of making errors is a more realistic approach than a formal Bayesian decision theoretic approach in which meaningful specification of utilities is speculative.

Currently, late-phase/confirmatory trials which seek to change practice are required by regulators to demonstrate control of type I error, whether the trial is designed and analysed in the Bayesian or frequentist framework. Trialists tend to have more freedom in the design and analysis of exploratory trials which seek to gather evidence to maximise the chances that the considerable resources required to run a confirmatory trial are only invested in the most promising treatments. Exploratory trials tend to have more relaxed type I error control requirements and regulators may be more amenable to use of a fully Bayesian decision-theoretic approach for exploratory trials.

## Conclusion

If one wishes to demonstrate control of type I error in Bayesian adaptive designs that allow for early stopping for efficacy then adjustments to the stopping boundaries are usually required as the number of analyses increase. If the designs only allow for early stopping for futility then adjustments to the stopping boundaries may instead be required to ensure that power is maintained as the number of analyses increase. If one wishes to instead take a strict Bayesian view then type I errors could be ignored and the designs instead focus on the posterior probabilities of treatment effects of particular values.

## Supplementary information

**Additional file 1.** R code for Time-to-event example.

**Additional file 2.** Operating characteristics for binary outcome example.

## Data Availability

The data used in this study were generated via simulation. The R code for the time-to-event outcome case study is presented in Additional file 1. The FACTS files for the binary outcome case study may be available on request from the corresponding author (EG Ryan). Requests for documents will be assessed on their individual merits.

## Abbreviations

RCT: Randomized controlled trial
RR: Relative risk
EMA: European Medicines Agency
FDA: US Food and Drug Administration
MHRA: Medicines and Healthcare products Regulatory Agency
HR: Hazard ratio
BALTI-2: Beta Agonist Lung injury TrIal 2
ARDS: Acute Respiratory distress syndrome
FACTS: Fixed and Adaptive Clinical Trial Simulator
