# Supplementary material for: Do we need to adjust for interim analyses in a Bayesian adaptive trial design?
Source: BMC Med Res Methodol. 2020 Jun 10;20:150. doi: 10.1186/s12874-020-01042-7 (PMC7288484; doi:10.1186/s12874-020-01042-7)
Supplement: Supplementary file 1 — Additional file 1. R code for Time-to-event example. [file 12874_2020_1042_MOESM1_ESM.docx]

Additional File 1 – R code for Time-to-event example

# R code for HR example with no interim analyses

| # Operating Characteristics - Norm-Norm Posterior (Hazard Ratio) |
| --- |
| # E - Number of events |
| # accept_prob = probability required to be greater than the required response rate with e.g. 0.8 |
| # crit_rate = required response rate to be less than e.g. 1, 0.9 etc. |
| # prior_par = vector of parameters for norm(mean=a,var=b) prior distribution |
| # HR = Underlying true hazard ratio |
| # p0, p1 = underlying true survival rates for control and experimental arms respectively |
| # nsims = number of simulations |
|  |
| oc_HR <- function(p0 = 0.5, p1 = 0.5, E, HR = p1/ p0, accept_prob, crit_rate, prior_par=c(0,10000),nsims){ |
|  |
| prob <- c(rep(0, nsims)) |
|  |
| for(i in 1:nsims) { |
|  |
|  |
| HR_True <- HR |
|  |
| VarLHR = 4/E |
| LHR <- rnorm(1, mean = log(HR_True), sd = sqrt(VarLHR)) |
|  |
| Prior_Mean <- prior_par[1] |
| Prior_Var <- prior_par[2] |
|  |
| post_prob <- pnorm(log(crit_rate), mean = (Prior_Mean/Prior_Var + LHR/VarLHR) / (1/Prior_Var + 1/VarLHR), |
| sd = sqrt(1 / (1/Prior_Var + 1/VarLHR)), lower.tail = TRUE) |
|  |
| ifelse(post_prob >= accept_prob, prob[i]<- 1, prob[i]<-0) |
| } |
|  |
| Value <- 100 * sum(prob, na.rm = TRUE) / (nsims - sum(is.na(prob))) |
|  |
| return(Value) |
| }  # oc_HR(HR = 1.0, E = 200, accept_prob = 0.90, crit_rate = 1.0, nsims = 1000000) |

# R code for HR example with 1 interim analysis performed which allows early stopping for futility

| OC_HR_Int<- function(HR, E, prior_par = c(0, 10000), crit_val, nsims, pthresh1, pthresh2) { | |
| --- | --- |
|  |  |
|  | results1 <- rep(NA, nsims) |
|  | results2 <- rep(NA, nsims) |
|  |  |
|  |  |
|  |  |
|  | for(i in 1:nsims){ |
|  |  |
|  |  |
|  | VarLHR = 4/(E/2) |
|  | LHR <- rnorm(1, mean = log(HR), sd = sqrt(VarLHR)) |
|  |  |
|  | Prior_Mean <- prior_par[1] |
|  | Prior_Var <- prior_par[2] |
|  |  |
|  | prob1 <- pnorm(log(crit_val), mean = (Prior_Mean/Prior_Var + LHR/VarLHR) / (1/Prior_Var + 1/VarLHR), |
|  | sd = sqrt(1 / (1/Prior_Var + 1/VarLHR)), lower.tail = TRUE) |
|  |  |
|  | results1[i] <- ifelse(prob1 < pthresh1, 1, 0) |
|  |  |
|  | if(results1[i] == 0) { |
|  |  |
|  | VarLHR = 4/(E) |
|  | LHR <- rnorm(1, mean = log(HR), sd = sqrt(VarLHR)) |
|  |  |
|  | Prior_Mean <- prior_par[1] |
|  | Prior_Var <- prior_par[2] |
|  |  |
|  | prob2 <- pnorm(log(crit_val), mean = (Prior_Mean/Prior_Var + LHR/VarLHR) / (1/Prior_Var + 1/VarLHR), |
|  | sd = sqrt(1 / (1/Prior_Var + 1/VarLHR)), lower.tail = TRUE) |
|  |  |
|  | results2[i] <- ifelse(prob2 > pthresh2, 1, 0) |
|  |  |
|  | } |
|  |  |
|  | } |
|  |  |
|  |  |
|  | return(paste0('Probability of stopping interim = ', 100*(sum(results1)/nsims) , |
|  | ' Probability of Go final \| passed interim = ', |
|  | 100* (sum(results2, na.rm = T) / (nsims - sum(is.na(results2)))), |
|  | ' Probability of Go final = ', 100 * (sum(results2, na.rm = T) / nsims))) |
|  |  |
|  | } |

#OC_HR_Int(HR = 1.0, E = 200, crit_val = 1.0, nsims = 1000000, pthresh1 = 0.50, pthresh2 = 0.90)

# R code for HR example with 1 interim analysis performed which allows early stopping for efficacy

| OC_HR_Int_Sup<- function(HR, E, prior_par = c(0, 10000), crit_val, nsims, pthresh1, pthresh2) { | | |  |
| --- | --- | --- | --- |
|  | | |  |
|  | results1 <- rep(NA, nsims) |  |  |
|  | results2 <- rep(NA, nsims) |  |  |
|  |  |  |  |
|  | for(i in 1:nsims){ |  |  |
|  |  |  |  |
|  |  |  |  |
|  | VarLHR = 4/(E/2) |  |  |
|  | LHR <- rnorm(1, mean = log(HR), sd = sqrt(VarLHR)) |  |  |
|  |  |  |  |
|  | Prior_Mean <- prior_par[1] |  |  |
|  | Prior_Var <- prior_par[2] |  |  |
|  |  |  |  |
|  | prob1 <- pnorm(log(crit_val), mean = (Prior_Mean/Prior_Var + LHR/VarLHR) / (1/Prior_Var + 1/VarLHR), |  |  |
|  | sd = sqrt(1 / (1/Prior_Var + 1/VarLHR)), lower.tail = TRUE) |  |  |
|  |  |  |  |
|  | results1[i] <- ifelse(prob1 > pthresh1, 1, 0) |  |  |
|  |  |  |  |
|  | if(results1[i] == 0) { |  |  |
|  |  |  |  |
|  | VarLHR = 4/(E) |  |  |
|  | LHR <- rnorm(1, mean = log(HR), sd = sqrt(VarLHR)) |  |  |
|  |  |  |  |
|  | Prior_Mean <- prior_par[1] |  |  |
|  | Prior_Var <- prior_par[2] |  |  |
|  |  |  |  |
|  | prob2 <- pnorm(log(crit_val), mean = (Prior_Mean/Prior_Var + LHR/VarLHR) / (1/Prior_Var + 1/VarLHR), |  |  |
|  | sd = sqrt(1 / (1/Prior_Var + 1/VarLHR)), lower.tail = TRUE) |  |  |
|  |  |  |  |
|  | results2[i] <- ifelse(prob2 > pthresh2, 1, 0) |  |  |
|  |  |  |  |
|  | } |  |  |
|  |  |  |  |
|  | } |  |  |
|  |  |  |  |
|  | return(paste0('Probability of stopping Superiority (interim) = ', 100*(sum(results1)/nsims) , |  |  |
|  | ' Probability of stopping Superiority (final) \| !Stop interim = ', |  |  |
|  | 100* (sum(results2, na.rm = T) / (nsims - sum(is.na(results2)))), |  |  |
|  | ' Probability of superiority overall = ', 100 * ((sum(results2, na.rm = T) + sum(results1))/ nsims))) |  |  |
|  |  |  |  |
|  | } |  |  |

#OC_HR_Int_Sup(HR = 0.7, E = 200, crit_val = 1.0, nsims = 1000000, pthresh1 = 0.90, pthresh2 = 0.90)
