# Supplementary material for: Do we need to adjust for interim analyses in a Bayesian adaptive trial design?
Source: BMC Med Res Methodol. 2020 Jun 10;20:150. doi: 10.1186/s12874-020-01042-7 (PMC7288484; doi:10.1186/s12874-020-01042-7)
Supplement: Supplementary file 2 — Additional file 2. Operating characteristics for binary outcome example. [file 12874_2020_1042_MOESM2_ESM.docx]

**Additional File 2 – Operating characteristics for binary outcome example**

**Table A2. Bayesian sequential designs explored for the binary outcome example and their operating characteristics**

| Number of interim analyses | Timing of interim (number of patients recruited) | Early stopping decision to be assessed | Type I error | Power |
| --- | --- | --- | --- | --- |
| 0 | NA | NA | 0.0204 | 0.8966 |
| 1 | 667 | Efficacy | 0.0232 | 0.9001 |
| 2 | 445 890 | Efficacy | 0.0258 | 0.8977 |
| 3 | 334 667 1001 | Efficacy | 0.0254 | 0.8961 |
| 4 | 267 534 801 1068 | Efficacy | 0.0261 | 0.8976 |
| 5 | 223 445 667 890 1112 | Efficacy | 0.0291 | 0.8956 |
| 6 | 191 382 572 763 953 1144 | Efficacy | 0.0274 | 0.8984 |
| 7 | 167 334 501 667 834 1001 1168 | Efficacy | 0.0279 | 0.9014 |
| 8 | 149 297 445 593 742 890 1038 1186 | Efficacy | 0.0272 | 0.8941 |
| 9 | 134 267 401 534 667 801 934 1068 1201 | Efficacy | 0.0288 | 0.8961 |
| 10 | 122 243 364 486 607 728 849 971 1092 1213 | Efficacy | 0.0295 | 0.8959 |
| 25 | 52 103 154 206 257 308 360 411 462 514 565 616 667 719 770 821 873 924 975 1027 1078 1129 1181 1232 1283 | Efficacy | 0.0303 | 0.8943 |
| 50 | 27 53 79 105 131 157 184 210 236 262 288 314 341 367 393 419 445 471 497 524 550 576 602 628 654 681 707 733 759 785 811 838 864 890 916 942 968 994 1021 1047 1073 1099 1125 1151 1178 1204 1230 1256 1282 1308 | Efficacy | 0.0333 | 0.8918 |
|  |  |  |  |  |
| 0 | NA | NA | 0.0204 | 0.8966 |
| 1 | 667 | Futility | 0.0188 | 0.8863 |
| 2 | 445 890 | Futility | 0.0195 | 0.8711 |
| 3 | 334 667 1001 | Futility | 0.0188 | 0.8593 |
| 4 | 267 534 801 1068 | Futility | 0.0191 | 0.8484 |
| 5 | 223 445 667 890 1112 | Futility | 0.0187 | 0.8354 |
| 6 | 191 382 572 763 953 1144 | Futility | 0.0184 | 0.8247 |
| 7 | 167 334 501 667 834 1001 1168 | Futility | 0.0153 | 0.8195 |
| 8 | 149 297 445 593 742 890 1038 1186 | Futility | 0.0151 | 0.8074 |
| 9 | 134 267 401 534 667 801 934 1068 1201 | Futility | 0.017 | 0.7987 |
| 10 | 122 243 364 486 607 728 849 971 1092 1213 | Futility | 0.0164 | 0.7907 |
| 25 | 52 103 154 206 257 308 360 411 462 514 565 616 667 719 770 821 873 924 975 1027 1078 1129 1181 1232 1283 | Futility | 0.0126 | 0.7113 |
| 50 | 27 53 79 105 131 157 184 210 236 262 288 314 341 367 393 419 445 471 497 524 550 576 602 628 654 681 707 733 759 785 811 838 864 890 916 942 968 994 1021 1047 1073 1099 1125 1151 1178 1204 1230 1256 1282 1308 | Futility | 0.0116 | 0.6608 |
|  |  |  |  |  |
| 0 | NA | NA | 0.0204 | 0.8966 |
| 1 | 667 | Efficacy or futility | 0.0217 | 0.8877 |
| 2 | 445 890 | Efficacy or futility | 0.0242 | 0.8732 |
| 3 | 334 667 1001 | Efficacy or futility | 0.0231 | 0.8602 |
| 4 | 267 534 801 1068 | Efficacy or futility | 0.0236 | 0.8496 |
| 5 | 223 445 667 890 1112 | Efficacy or futility | 0.0246 | 0.8377 |
| 6 | 191 382 572 763 953 1144 | Efficacy or futility | 0.0234 | 0.8271 |
| 7 | 167 334 501 667 834 1001 1168 | Efficacy or futility | 0.0232 | 0.8234 |
| 8 | 149 297 445 593 742 890 1038 1186 | Efficacy or futility | 0.0226 | 0.8089 |
| 9 | 134 267 401 534 667 801 934 1068 1201 | Efficacy or futility | 0.0253 | 0.8015 |
| 10 | 122 243 364 486 607 728 849 971 1092 1213 | Efficacy or futility | 0.0248 | 0.7926 |
| 25 | 52 103 154 206 257 308 360 411 462 514 565 616 667 719 770 821 873 924 975 1027 1078 1129 1181 1232 1283 | Efficacy or futility | 0.0221 | 0.707 |
| 50 | 27 53 79 105 131 157 184 210 236 262 288 314 341 367 393 419 445 471 497 524 550 576 602 628 654 681 707 733 759 785 811 838 864 890 916 942 968 994 1021 1047 1073 1099 1125 1151 1178 1204 1230 1256 1282 1308 | Efficacy or futility | 0.0243 | 0.6573 |
